# Editorial: Gynecological cancer prevention in women with inherited genetic risk

**DOI:** 10.3389/fonc.2026.1978419

**Published:** 2026-09-18

**Authors:** Robert Fruscio, Anton A. Buzdin

**Affiliations:** 1 Department of Medicine and Surgery, University of Milano-Bicocca, Milan, Italy; 2 UO Gynecology, Fondazione IRCCS San Gerardo dei Tintori, Monza, Italy; 3 Group for Genomic Regulation of Cell Signaling Systems, Shemyakin-Ovchinnikov Institute of Bioorganic Chemistry, Moscow, Russia

**Keywords:** BRCA 1/2 mutation carriers, genetic predisposition, genetic testing, gynecological cancer, prevention

The prevention of gynecological malignancies in women with inherited susceptibility has moved from a largely family-history-based discipline to one increasingly shaped by multigene testing, molecular pathology, and individualized risk estimates. The main challenge is no longer simply identifying a pathogenic variant, but converting genetic information into timely, acceptable, and equitable interventions while preserving fertility, sexual health, and long-term quality of life. The eight contributions to this Research Topic illustrate different components of this transition from genetic diagnosis to precision prevention and highlight an increasingly important reality: advances in genetic discovery must now be matched by advances in implementation.

A first major theme is access to genetic testing and translation of genetic information into care. Duranti et al. provide a broad framework integrating carrier identification, counseling, surveillance, chemoprevention, and risk-reducing surgery. However, availability of genetic knowledge does not necessarily translate into implementation. In Chinese women with epithelial ovarian cancer, Wang et al. report a germline testing uptake of 64.9%, despite almost two-thirds of participants having low awareness. Testing was associated with higher income, family history, and greater genetic knowledge, while pathogenic variants were identified in 19.6% of those tested. These findings highlight the gap between recommendations and real-world practice. Mainstreamed testing, adequate reimbursement, culturally appropriate education, and systematic cascade testing are essential if identification of a pathogenic variant in one patient is to become prevention for an entire family.

Molecular pathology is increasingly central to hereditary cancer prevention. Tumor morphology and molecular characteristics may provide the first indication of an underlying syndrome. Universal mismatch-repair testing in endometrial cancer, pathology-driven referral for germline evaluation, and reflex molecular testing illustrate how pathology can become an active component of hereditary cancer pathways. Integrated reports combining histological, immunohistochemical, and relevant molecular information may reduce missed opportunities for genetic assessment. Molecular classifiers, including MMR and POLE status in endometrial cancer, further illustrate the convergence of pathology, molecular oncology, and individualized management, although their implications for hereditary risk assessment differ.

A second major theme concerns risk-reducing interventions and reproductive choices. Ovarian cancer screening remains ineffective, and risk-reducing salpingo-oophorectomy remains central for appropriately selected women at sufficiently high inherited risk. Preventive decisions, however, must consider not only gene-specific penetrance and age-related risk, but also reproductive plans, premature menopause, sexual health, quality of life, and individual preferences. Salpingectomy with delayed oophorectomy represents an attractive alternative, but definitive evidence regarding its oncological safety is still awaited. Liu et al. similarly illustrate the balance between prevention and reproductive autonomy in Lynch syndrome-associated endometrial cancer. Carefully selected fertility-sparing treatment may allow pregnancy, including through assisted reproduction, while preimplantation genetic testing for monogenic disorders may reduce transmission of mismatch-repair variants. These approaches require non-directive counseling, careful surveillance, and definitive surgery after completion of childbearing.

Rare hereditary syndromes and unusual pathological findings further emphasize that prevention often begins with recognition. The FH-deficient uterine leiomyoma reported by Wang et al. highlights the role of FH/2SC immunohistochemistry and genetic counseling when hereditary leiomyomatosis and renal cell carcinoma is suspected. Reports by Li et al. and Wu et al. of gastric-type endocervical adenocarcinoma in Peutz-Jeghers syndrome similarly underscore the association between STK11-related disease and an aggressive, HPV-independent cervical malignancy. Tumor morphology, immunophenotype, syndromic physical findings, and family history may therefore all represent entry points to genetic assessment and appropriate surveillance.

Other contributions illustrate the expanding complexity of genetic information. Reports of cervical cancers harboring BRCA1 variants and rare BRCA1/BRIP1 or BRCA1/BRCA2 double heterozygosity may provide insights into genotype-phenotype relationships, but small case series cannot establish causality, penetrance, or new screening indications. Furthermore, expanded multigene testing increasingly identifies variants of uncertain significance, creating challenges for counseling and potentially generating anxiety or inappropriate interventions if findings are overinterpreted. International registries, functional studies, and prospective genotype-phenotype analyses remain essential.

Important limitations and disparities persist. Prospective evidence is limited for several preventive strategies, while the psychological burden of inherited cancer risk, repeated surveillance, reproductive decisions, and irreversible preventive surgery remains insufficiently addressed. Cost and access are major barriers, with inequalities extending beyond income to rural populations, ethnic and cultural groups, geographic regions, and low-resource healthcare systems. Precision prevention cannot succeed if its benefits remain concentrated in highly specialized centers.

Emerging technologies may further refine prevention. Polygenic risk scores could complement monogenic risk assessment, long-read sequencing may improve detection of complex variants, and liquid biopsy and digital pathology may contribute to earlier recognition and risk stratification. Artificial intelligence may support variant interpretation and aspects of genetic counseling, although rigorous validation and attention to bias, privacy, and preservation of patient-centered decision-making will be essential.

Taken together, these contributions suggest that the major challenge in hereditary gynecological cancer prevention is increasingly implementation rather than discovery. Genetic knowledge has expanded rapidly, but its preventive value depends on equitable access to testing, reliable variant interpretation, integrated molecular pathology, multidisciplinary counseling, evidence-based risk reduction, cascade testing, and long-term follow-up. Future research must therefore extend beyond discovery to implementation science, prospective evaluation of preventive strategies, health economics, psychosocial outcomes, and disparities. The promise of precision prevention will ultimately be realized not simply when inherited risk can be identified, but when that knowledge can be consistently transformed into coordinated, equitable, and person-centered care for patients and their families.

